# Revisiting sweat chloride test results based on recent guidelines for diagnosis of cystic fibrosis

**DOI:** 10.1016/j.plabm.2018.01.001

**Published:** 2018-01-03

**Authors:** Jayson V. Pagaduan, Mahesheema Ali, Michael Dowlin, Liye Suo, Tabitha Ward, Fadel Ruiz, Sridevi Devaraj

**Affiliations:** Department of Pathology & Immunology, Baylor College of Medicine, Department of Pathology and Immunology, Texas Children's Hospital, Houston, TX 77030, USA

**Keywords:** Cystic fibrosis, Updated guideline, Sweat chloride, Pilocarpine iontophoresis

## Abstract

**Objectives:**

Recent sweat chloride guidelines published by the Cystic Fibrosis Foundation changed the intermediate sweat chloride concentration range from 40–59 mmol/L to 30–59 mmol/L for age > 6 months. We wanted to know how this new guideline would impact detection of cystic fibrosis among patients who previously had sweat tests done at Texas Children's Hospital.

**Methods:**

We revisited sweat chloride test results (n = 3012) in the last 5 years at Texas Children's Hospital based on the new guidelines on diagnosis of cystic fibrosis from the Cystic Fibrosis Foundation.

**Results:**

We identified 125 patients that would be reclassified in the intermediate sweat chloride value with the new guidelines that were classified as “unlikely to have CF” in the previous guidelines. 8 (32%) patients with CFTR gene testing were positive for CFTR gene mutation(s). 4 (50%) of these patients were identified to have 2 CFTR mutations. One had variant combination that was reported to cause CF but all were diagnosed with CFTR-related metabolic syndrome.

**Conclusion:**

Our findings concur with the new CF diagnosis guidelines that changing the intermediate cut-off to 30–59 mmol/L sweat chloride concentration in combination with CFTR genetic analysis enhances the probability of identifying individuals that have risk of developing CF or have CF and enables for earlier therapeutic intervention.

## 1 Introduction

Cystic fibrosis (CF) is an autosomal recessive disorder that may be present in individuals with mutations in the cystic fibrosis transmembrane conductance regulator (CFTR) gene. CFTR protein is mainly involved in chloride ion channel that is involved in producing sweat, saliva, tears, mucus and digestive enzymes [1]. CFTR gene testing is also available to determine the type of CFTR mutation present especially when the sweat chloride test falls within the intermediate range. However, different genotypes present varying clinical manifestations from healthy to diseased. Therefore, sweat chloride testing using quantitative pilocarpine iontophoresis is the standard for CF diagnosis [2]. It is important to diagnose CF early to be able to render proper therapy to affected individuals, improve their quality of life and ultimately avoid morbidity.

Recently, the Cystic Fibrosis Foundation published updated guidelines for diagnosis of cystic fibrosis and lowered the intermediate range for sweat chloride concentration to 30–59 mmol/L for all populations, from previous 40–59 mmol/L for age > 6 months [3]. We revisited previous sweat chloride test results performed at Texas Children's Hospital in the last 5 years to determine the impact of the new guidelines in CF diagnosis.

## 2 Methods

The protocol was IRB approved. The detailed procedure of sweat collection and analysis was previously published [4], [5]. Briefly, trained technologists from Texas Children's Hospital performed quantitative pilocarpine iontophoresis and sweats were collected using macroduct sweat collectors. Sweat chloride measurement were done using Wescor ChloroChek Model 3400.

Sweat tests (n = 3012) done in the last 5 years at Texas Children's Hospital were gleaned for sweat chloride test results that fall within 30–39 mmol/L and individuals who were > 6 months of age. All sweat chloride testing were done bilaterally but not all tests produced adequate sample volume for testing. Therefore, all patients age > 6 months and with at least one chloride testing with value that falls within 30–39 mmol/L were included in the analysis. All patients with at least one sweat chloride result > 40 mmol/L (n = 66) were not included in the analysis because these would have been included in the previous range of 40–60 mmol/L for intermediate results [6].

Then, we looked at the patient electronic medical record to determine if they had CFTR gene testing done and if individuals were diagnosed with CF. If CFTR mutation was reported, we searched the Clinical and Functional Translational of CFTR website to determine the significance of the mutation.

## 3 Results and discussion

A total of 3012 sweat tests were performed in the last 5 years. 2097 of these tests were from 1872 patients with age > 6 months while 1015 tests were from 833 patients with age < 6 months and not included in further analysis.

Table 1 summarizes the comparison of sweat chloride testing results by age group between the old and new CF guidelines. 125 more patients were categorized in the intermediate range when the new guideline for CF diagnosis was applied to previous patients resulting in a total of 191 subjects who were now in the intermediate range. The reason we chose 30–39 mmol/L is because, using the previous guidelines, > 40 would have been picked up as being in the intermediate range for CF. Thus, out of the 125 new patients classified as intermediate for CF based on the new guidelines, our investigations showed that 25 (20%) had CFTR gene testing results recorded (see Table 2).

**Table 1:** Sweat chloride test results based on the old and new CF diagnosis guidelines.

***Old guideline (Age > 6 Months)***			
**Sweat [Cl] mmol/L**	**<40**	**40–59**	**> 60**
**Number of Patients**	**1711**	**66**	**95**
***New guideline (Age > 6 Months)***			
**Sweat [Cl] mmol/L**	**<30**	**30–59**	**> 60**
**Number of patients**	**1586**	**191**	**95**

**Table 2:** Prevalence of CFTR mutation among individuals with sweat chloride concentration 30–39 mmol/L and age > 6 months (n = 125).

**CFTR gene testing results available**	**Total**	**With CFTR gene mutation**
Yes	25	8
No	100	–

We also reviewed the electronic medical records of these 125 patients and searched for CFTR gene testing results, mutation genotype and physician's diagnosis. Table 3 summarizes the medical history of the 8 patients identified to have CFTR gene mutation(s). New born screening (NBS) identified 3 patients with CFTR gene mutations. When NBS is found positive for CFTR mutation, sweat chloride test is performed to verify CF diagnosis. The other patients presented with various symptoms such as respiratory problems and pancreatitis, which are commonly manifested in CF patients.

**Table 3:** Medical history of patients with CFTR gene mutation(s).

**Patient**	**Medical history**
1	NBS positive for CFTR mutation
2	NBS positive for CFTR mutation
3	NBS positive for CFTR mutation
4	Acute pancreatitis
5	Mild persistent asthma without complications, allergic rhinitis
6	Recurrent pancreatitis
7	Recurrent acute pancreatitis
8	Chronic cough and nasal congestion

Table 4 summarizes the prevalence of CFTR mutations among individuals with sweat chloride concentration 30–39 mmol/L and with age > 6 months. 8 (32%) patients had CFTR gene mutation(s) detected. 4 of them had 2 CFTR mutations with one of the mutations identified as phenylalanine deletion (F508del), which is the most common mutation that is responsible for ~ 70% of CFTR mutation with CF worldwide [7]. All four were diagnosed with CFTR-related metabolic syndrome (CRMS). Even though there were no definitive CF patients identified among these individuals, it is important to remember that most CRMS individuals will remain healthy but have potential to develop CF [8]. Furthermore, one patient had CFTR mutation combination of F508del and R117C but was still not diagnosed as CF, even though this variant combination has been reported in The Clinical and Functional Translation of CFTR (CFTR2) database as causing CF.

**Table 4:** CFTR mutation genotypes, sweat test results and corresponding clinical diagnosis.

**Patient**	**CFTR genotype**	**Average sweat test results (mmol/L)**	**Clinical diagnosis**	**CFTR2 note**
1	F508del, R117H (7T)	35,38, and 42.5	CRMS	Varying consequences
2	F508del, TG12-5T	33.5	CRMS	Varying consequences
3	F508del, R117C	27.5 and 37	CRMS	Causes CF
4	F508del, G576A	37	CRMS	Does not cause CF
5	R117H (7T)	28	CF mutation carrier	Varying Consequences
6	861delT	23.5 and 31.5	CF mutation carrier	NA
7	TG(11-5T)	34	CF mutation carrier	NA
8	C53+9GT	34 and 34.5	CF mutation carrier	NA

Other patients had one CFTR mutation identified. One patient had R117H(7T) mutation. Three other CFTR mutations detected were assigned as (TG)11–5T, 861delT and C53+9GT were not previously reported in the CFTR2 database [9]. 4 of the 8 patients had at least 2 repeat sweat tests. All repeat sweat tests were done at least 1 year after the previous test. Interestingly patient 1 sweat tests were trending up though still within the intermediate range which warrants regular check-up of the patient by the primary care physician or CF specialist.

The spectrum of clinical symptoms associated with CFTR genotypes emphasizes the importance of scrutinizing the clinical symptoms of the patient to increase that chance of early detection, and regular monitoring of individuals with CFTR mutations. Table 3 summarizes the CFTR mutation combination, average sweat test results, the clinician's diagnosis and observation described in the CFTR2 database.

## 4 Conclusion

Revisiting previous sweat chloride tests based on the new guidelines for CF diagnosis resulted in re-classification of 125 patients from unlikely CF diagnosis to intermediate sweat chloride. These 125 patients had sweat chloride values that fall within 30–39 mmol/L. Only 25 individuals (20%) were tested for CFTR gene mutation. 4 of 25 (16%) were diagnosed with CRMS. CRMS individuals may remain healthy but regular follow-up is recommended to ensure healthy status and that proper therapy is given if condition worsens. Genetic testing for CFTR mutation is important to determine the presence or absence of disease-causing mutations especially for individuals with intermediate sweat test results. However, CFTR mutations present varying clinical consequences making it difficult to diagnose based on genetic testing alone. Therefore, it is important to report newly identified mutations and the corresponding clinical signs to improve the understanding of the spectrum of CF conditions. The CFTR2 database is an excellent repository of CFTR gene mutations available for the public. Our findings agree with the new CF diagnosis guideline of lowering the intermediate cut-off values from 40–59 mmol/L to 30–59 mmol/L and performing genetic analysis to determine the presence of deleterious CFTR gene mutation to increase the probability of identifying individuals with CF and enable health providers to give appropriate therapy to help improve the quality of life of the affected individuals.
